# Analysis of Contemporary Violin Recordings of 19th Century Repertoire: Identifying Trends and Impacts

**DOI:** 10.3389/fpsyg.2018.02233

**Published:** 2018-11-27

**Authors:** Eitan Ornoy, Shai Cohen

**Affiliations:** ^1^Faculty of Music Education, Levinsky College of Education, Tel Aviv, Israel; ^2^Department of Music, Bar-Ilan University, Ramat Gan, Israel

**Keywords:** violin recordings, performance analysis, historically informed performance (HIP), mainstream performance (MS), 19th century performance

## Abstract

The study of violin recordings as evidence of interpretation and performance approach has been quite extensive throughout recent decades. Findings such as the limited use of vibrato in early recordings, the relative stabilization of tempo and rhythm characteristic of mid-20th century violin playing, or the effect of ‘historically informed performance’ (HIP) on ‘mainstream’ (MS) violinists performing early music repertoire, are fundamental in the identification of prevailing norms of practice and changes of style that have occurred over time. However, compared to the considerable amount of research conducted on 20th century playing, studies focusing on 21st century violin performances are still quite limited, and are mostly based on recordings of J. S. Bach’s sonatas and partitas for solo violin. This paper examines contemporary violin recordings made in recent decades (1999–2017). It aims to explore the most current trends in early 21st century violin performance practices and identify the impact of HIP principles on the new generation of players. Unlike most previous research, it focuses on recordings made to 19th century repertoire, thus enlarging the spectrum to include performance analysis of a relatively untouched field and enabling examination of the degree and manner of incorporating 19th century performance attributes (e.g., utilization of rhythmic unevenness, portamento or harmonics) into contemporary praxis. Results suggest an extensive blend of stylistic approaches among currently active violinists, putting into question the relevance of the traditional distinction made between HIP and MS performance styles when it comes to current performance vogue. Incorporating 19th century performance devices while playing 19th century music was traced in varying degrees. Findings are suggested as being part of a general, quasi-postmodern quest for pluralism and elimination of hierarchical classifications, representing an era of ‘over-choice’ environment.

## Introduction

The study of violin recordings as evidence of interpretation and performance approach has been quite extensive throughout recent decades. The scope of documented material has greatly increased over the last century, not only in the number of performers recording their craft but also in the range of recorded repertoire, which includes music of various periods and multiple recordings of the same work by any single performer.

Over the years, a wide palette of personal characteristics, distinctiveness, and individual peculiarities was found in the practice of prominent figures active throughout the entire 20th century. Specific approaches to phrasing, dynamics, bowing, tempo, rhythmic choices, vibrato, portamento, fingerings, and the like found to blur what appeared to be period modes. Such remarkable diversity of nuances was mostly traced in studies focusing on J. S. Bach’s six sonatas and partitas for solo violin (e.g., Pulley, 2002; Fabian, 2005; Ornoy, 2008; Cheng and Chew, 2008; Fabian and Ornoy, 2009; Dimov, 2010; Sretenovic and Adamovic, 2012; Gyulnazarova, 2013; Sarlo, 2015), but was also reported in recordings of several additional pieces written for either the violin (e.g., Levy, 2001; Katz, 2003; Lee, 2006; Stijn, 2009; Leech-Wilkinson, 2009) or other stringed instruments (e.g., Turner, 2004; November, 2010, 2011; Milisavljevic, 2011; Hong, 2014).

Despite a variety of styles and the seemingly overwhelming divergence and individual idiosyncrasies revealed among performances, several period characteristics were deduced:

Early recordings, grossly encompassing the period between 1898 and 1930^1^, were found to exhibit extensive use of portamento, limited vibrato (its use gradually increased during the 1920s), considerable utilization of harmonics, and a general inclination toward tempo fluctuations and rhythmic unevenness. The latter, apparent through extended use of inner phrase modifications (accelerando–rallentando), agogic accents (i.e., the lengthening or shortening of notes) or ‘melodic rubato’ (i.e., the use of a flexible melody upon a solid and strict-pulsed accompaniment) was found to be a prominent factor emerging from an overall rhetorical approach toward the musical language. Recordings made during these decades are extrapolated as representative of 19th century performance traditions (Philip, 1993, 2004; Brown, 2003; Katz, 2003; Milsom, 2003; Leech-Wilkinson, 2009).

Recordings of the intermediate period, encompassing the period between, say, 1930 and 1970, tend to exhibit more constraint with regard to inner- phrases tempo alterations and to avoid rhythmic vacillations that might sharply stand out from the written score. However, overall tempo choices were found to vary widely, and performers have by no means abandoned the praxis of tempo and rhythmic modifications. Vibrato was found to be highly practiced in various manners of execution (although still used quite sparingly at times) with regards to width, speed, onset, and duration. While unequivocally found to be displayed on a much broader scale from the 1930s onward, a decrease of vibrato width and rate was reported to have emerged from the 1960s, suggested as being connected to a broader palette of expressive devices (Turner, 2004; Dimov, 2010). The practice of portamento as an intentional means of expressivity (rather than as a technical device used during position shifts) has largely declined, although never vanished. Harmonics were considerably reduced, together with avoiding open strings by fingering the note on its adjacent lower string. Articulation, while certainly varied in its manifestation, has tended toward considerable use of tenuto, seamless bowings, long and smooth legato lines used alongside even portato strokes, and clear-cut, articulated enunciation of staccato notes boosting brilliant virtuosic display and superb technical command (Stowell, 1998; Katz, 2006; Fabian, 2006, 2009; Ornoy, 2006, 2008; Fabian and Ornoy, 2009; Leech-Wilkinson, 2009; Sarlo, 2015).

During the final decades of the 20th century, the rise of the “HIP” movement had an overwhelming impact on violin performance aesthetics. The aspiration to reconstruct a multitude of seemingly bygone performance traditions when playing early repertoire has led to an increased awareness and legitimization of the numerous possibilities for realization of the written text. Questions posed throughout the years have certainly disputed the plausibility of reconstructing bygone performance traditions amidst challenging the very quest for historical accuracy, yet all in all the HIP approach still serves to reflect fidelity to past performance aesthetics (Taruskin, 1995; Butt, 2002; Walls, 2003; Haynes, 2007; Kuijken, 2013).

Most HIP attempts focused on incorporating performance features of pre-1800 repertoire, for which several characteristics emerged. These include the utilization of Baroque violin coupled with gut strings and assorted bows varied according to the specific style and period of composition, employment of particular dynamics attributes such as the use of Messa di Voce (which involves a gradual crescendo while sustaining a single pitch), explicit utilization of tempo and rhythmic alterations, frequent arpeggiation of chords, use of Baroque pitch tuning, sparse usage of vibrato, observance of original notated bowings through the use of a critical edition, the use of open strings assisted by fingerings adhering to the lower positions, or the employment of a wide spectrum of articulation. Together with the focus on early music repertoire and a significant engagement with HIP specialists throughout ones’ long years of artistic training, they seem to grossly define a violinist’s affiliation with the HIP style. On the other hand, inclination toward features that are more in line with the intermediate recording period are considered representatives of the ‘mainstream’ (MS) performance characteristics (Fabian, 2005, 2015; Ornoy, 2006, 2008, 2016; Leech-Wilkinson, 2009).

However, since its rise, the effect of HIP on MS violinists’ recording early music repertoire has gradually become all-embracing: similar choices regarding articulation devices, dynamic nuances, means of vibrato, tempo and rhythm manifestations, ornamentation, phrasing or fingerings were found to suggest mutual influences and shared canonical interpretations, and it seems clear that violinists have turned to a wide palette of performance features regardless of their seeming association to any one ‘school’ (Ornoy, 2006, 2008, 2016; Fabian, 2009, 2013, 2015; Fabian and Ornoy, 2009; Dimov, 2010; Liebman et al., 2012).

Moreover, while most studies have focused on recordings comprised of Baroque repertoire, several investigations witnessed the manifestation of performance features associated with the HIP style in the interpretations of 19th century repertoire. This emerges from the trend toward shallow vibrato and an increase in the use of portamento found in recordings of Beethoven and Brahms violin concertos and recordings of Beethoven string quartets, all made toward the final decades of the last century (Katz, 2003; Turner, 2004; Leech-Wilkinson, 2009), or the “urge toward innovation” using a variety of bow strokes and articulation devices found among the newer generation of string quartets (November, 2011).

Recent inquiries into aspects of 19th century style (such as the newly established TCHIP research project based at Oxford University^2^) mark the propagation of HIP approaches and methods, which nowadays are gradually starting to include investigation of 19th century performance traditions. A case in point could be traced within the substantial amount of work dedicated to Joachim’s recorded output. Being by far the most influential violinist of the 19th century German violin school, as well as one of the oldest musicians captured on records, Joachim’s recordings attract researchers who have taken up his playing as a representation of late 19th century German music aesthetics. His recordings are viewed as the live illustration of a rich heritage, which also consists of his monumental *Violinschule* (written in collaboration with Andreas Moser) and various editions made of key works of the violin repertoire. Such a rare combination of sources enables a unique and inclusive examination of both his artistic ideals and his overall Zeitgeist.

Scholars who have studied Joachim’s recordings have indicated his scarce use of vibrato (it being mostly displayed on long notes), occasional use of portamento, extensive use of tempo rubato as a means of emphasizing pivotal notes of structural significance, and a general feeling of rhythmic irregularity used for delineating inner-line phrases and emotional intensity. In his writings Joachim was quite resolute in opposing over-use of both vibrato and portamento (Brown, 2003; Kennaway, 2011). On the other hand, he is reported to have exhibited a variety of styles set out in accordance with the specific repertoire performed. Such was his almost eccentric use of rubato and extensive use of vibrato in his arrangements to Brahms’ Hungarian dances, as opposed to the relative stability of pace and the sparse and shallow vibrato found in his recording of Bach’s G minor Prelude. Another example is his moderate show of portamento in Bach’s Prelude, as opposed to its extensive use in the recording made of Joachim’s Romance in C (Brown, 2003; Ornoy, 2008; Leech-Wilkinson, 2009; Stijn, 2009).

An examination of recordings made by Joachim’s pupils (Karl Klinger, Marie Soldat-Roeger, Adela Fachiri, Maud Powell) made it possible to come up with a broader outlook on what could be considered the Joachim-Berlin school, whose main characteristics are the use of agogic accents at the beginning of a bar, salient rubato exhibited by playing the second half of the bar lighter and faster than its beginning via a more springy and bouncy articulation, a somewhat ‘tight’ finger vibrato used quite scantly, inclination toward broad tenuto bowings, the use of slow portamento, and the strong feeling at times of an almost eccentric rhythmic volatility. Its influence is said to have reached the playing of violinists not directly related to Joachim’s teachings, such as Arnold Rosé, Joseph Suk, Lucien Capet, and Emil Hauser (Philip, 1993, 2004; Milsom, 2003, 2007; Fabian, 2006).

However, compared to the wealth of research conducted on 20th century playing, studies focusing on 21st century violin performance are still quite limited, and are mostly based on recordings of the Bach solo violin set (e.g., Liebman et al., 2012; Fabian, 2013, 2015). On the whole, they continue to suggest that HIP performers integrate performance attributes characteristic of pre-1800 works more so than their MS peers, even though a high variance with regards to performance approaches and trends has been detected among the vast majority of players. Tempo choices were found to be dependent on personal preferences rather than connected to background parameters such as age, recording date or school classification. Vibrato has gradually become more varied over the last decades. A wide variety of bowing and articulation has been found among younger and HIP inspired players, who also portray shorter musical gestures by articulating concise units, as opposed to long-units phrasing found among older players.

Considering the substantial amount of investigations that have found wide implementation of HIP features in the playing of late 20th century violinists, it appears that HIP objectives have increasingly gained acceptance among contemporary players. However, most studies identifying HIP trends were conducted on late 20th century playing of pre-1800 repertoire. It remains unclear whether present-day violinists integrate attributes associated with 19th century performance traditions or whether new trends have developed, and to what extent.

## Aims

This study examines contemporary violin recordings made in recent decades (1999–2017). It aims to explore the most current trends in early 21st century violin performance practices, and to identify the impact of HIP principles on the new generation of players.

Unlike most previous research, it focuses on recordings made of 19th century repertoire, thus expanding the spectrum to include performance analysis of a relatively untouched field. The repertoire chosen for examination, representing two German composers from the beginning and late Romantic period (i.e., Beethoven and Brahms), enables a novel investigation of the degree and manner of incorporating 19th century performance attributes into contemporary praxis, as well as an observation of present-day playing trends.

### Hypotheses

The following predictions are postulated:

(1) Present-day violinists employ a wide palette of performance features and display diversity with regards to performance approaches and trends.
(2) Present-day violinists incorporate 19th century performance attributes while playing 19th century music.
(3) Present-day violinists distinguish between the manner of playing early (i.e., Beethoven) and late (i.e., Brahms) 19th century repertoire by employing diverse performance features.
(4) HIP performers integrate performance attributes characteristic of early music repertoire while playing early 19th century music more so than their MS peers.

## Materials and Methods

Twenty recordings of currently active violinists, produced during the last two decades (1999–2017), were analyzed for their manner of execution of various performance parameters, as well as for their correspondence to 19th century performance traditions.

Analysis was conducted for the following repertoire:

Beethoven Violin Sonata No. 6 in A Major, Op. 30, No. 1 (composed: 1802)

(a) Movement 1 (Allegro)
(b) Movement 2 (Adagio molto espressivo): bars 1–6
(c) Movement 3 (Allegretto con variazioni): Variation 4

Brahms Violin Sonata No. 1 in G Major, Op. 78 (composed: 1879)

(a) Movement 1 (Vivace ma non troppo)

The repertoire chosen for analysis was selected for the following reasons:

(1) Compositional date: both sonatas represent the beginning and late Romantic period.
(2) Recording scope: focusing on these two works, whose popularity has generated a large number of recordings, enables analyses of a wide collection of players and performance styles.
(3) Contour and layout: the various movements and segments selected for analysis highlight diverse performance features. For example: while Beethoven’s Sonata’s second movement exhibits long notes which are more apt for vibrato analysis, variation 4 of its third movement consists of multiple-stops (thus making it more fit for chords examinations), whereas the first movement’s layout serves more suitable for articulation and bowings scrutiny. The heterogenic nature of Brahms Sonata’s first movement as regards contour and formation enabled the selection of various sections from within the movement without the need for further analysis of its succeeding chapters.
(4) Pertinence: written during his transitional period, Beethoven’s sonata marks the dissolution of the composer’s predominant classical language and his groping for new means of dramatic expressions. As such, and taking to consideration its early compositional date, it aptly serves both HIP and MS renderings: while the former group mainly focuses on pre-1800 repertoire, up-to-date inspection of HIP praxis testifies to the growing penchant for Beethoven’s early output, most likely due to its proximity to 18th century music vernacular. As for the latter group, the sweeping popularity of Beethoven’s violin sonatas among MS performers is unequivocal, bringing about ample renditions of the work in hand.

Brahms’s sonata was composed in the wake of his violin concerto and presented complete to Joachim while both were working on the latter. Although irrelevant as a source for studying HIP performers, tracing performance features among current MS recordings of the sonata would reveal whether it is in compliance with late 19th century performance traditions in general, and with the so-called ‘Joachim-Berlin’ violin school in particular.

Table 1 shows HIP, MS and C19th performance characteristics gathered on the basis of previous studies made on the subject, coupled with the performance features analyzed and their related methods of investigation.

**Table 1:** List of performance features analyzed, style characteristics, and analysis method.

Performance feature	C19th performance characteristics, including ‘Joachim-Berlin’ school [JBS]	HIP performance characteristics	MS performance characteristics	Analysis method
Dynamics	No noted peculiarities	Use of Messa di Voce	Wide spectrum of dynamics	Examination of mean power (intensity) as found in selected notes, carried by sound waves per unit area expressed in dB versus time, including maximum, minimum, mean and deviation
Overall tempo	Wide options of overall tempo range	Wide options of overall tempo range	Wide options of overall tempo range	Calculation of the total length of selected movements, featured by both time units (expressed in seconds) and BPM
Tempo and rhythmic alterations	Rhythmic unevenness using means such as inner tempo fluctuations, agogic accents and melodic rubato. JBS: use of agogic accents at the beginning of a bar	Frequent tempo and rhythmic alterations	Stable tempo and rhythmic pronunciation	Calculation of the time duration of selected notes and measures, made to examine altered note values within the written score, including tempo fluctuations and the employment of inner phrase modifications (accelerando–rallentando), lengthening or shortening of notes (‘agogic accents’) and the use of ‘melodic rubato’
Multiple-stop progression	No noted peculiarities	Frequent arpeggiation of chords	Frequent use of broken-chords during multiple-stops progression	Examination of chord ratio and its manner of execution as found in selected measures
Intonation profile	No noted peculiarities	Low pitch tuning	Standard, high pitch tuning	Analysis of the mean deviation of the performed pitch as found in selected sampled measures, compared to equal temperament’s absolute frequency value (*A* = 440).
Vibrato	Limited vibrato. JBS: ‘tight’ finger vibrato used quite scantly	Sparse usage of vibrato	Extensive use of vibrato portrayed in various manners	Analysis of vibrato as found in selected notes, split into three ‘sub-features’: depth, speed and onset
Timbre	No noted peculiarities	Wide spectrum of timbre options	Wide spectrum of timbre options	Analysis of spectrum and envelope spectrum approximation and spectral component differences, expressed in sound pressure level (dB/Hz), extracted for the fundamental frequency and overtones of selected notes
Bowings and fingerings	Extensive use of portamento. Considerable utilization of harmonics. JBS: slow, yet extensive use of portamento in playing late Romantic-period repertoire.	Observance of original notated bowings; Frequent usage of open strings; Fingerings adhered to the lower positions	Sparse usage of portamento; General avoidance of harmonics and open strings	Examination of performed slurs and audible bow direction, as well as harmonics and position shifts that could be clearly detected in the selected excerpts
Articulation	JBS: broad tenuto bowings coupled with springy and bouncy articulation	Employment of a wide spectrum of articulation	Considerable use of tenuto bowings, smooth legato lines, even portato strokes and clear-cut enunciation of staccato notes	Investigation as to the use of tenuto, accents, spiccato, etc. found in selected excerpts


HIP violinists were extrapolated upon compliance to the following background factors:

(a) Utilization of period instruments in previous artistic enterprises
(b) Formal studies under the guidance of a HIP specialist

Table 2 displays the list of performers analyzed, their date of birth, recording date and label, style affiliation and performer’s general background. Note that due to its late compositional period, HIP performers (who specialize in pre-1800 repertoire) are missing from the Brahms sonata listing.

**Table 2:** List of analyzed recordings.

Performer	Birth	Recording	Label	Style affiliation	Performer’s general background
**Beethoven: Violin Sonata No. 6 in A Major, Op. 30, No. 1 (1802)**					
Corey Cerovsek (Paavali Jumppanen, Pf.)	1972	2006	Claves Records	MS	Former student of Joseph Gingold.
Isabelle Faust (Alexander Melnikov, Pf.)	1972	2009	Harmonia Mundi	HIP	Former student of Christoph Poppen and Dénes Zsigmondy. Previous artistic enterprises include performance with period violins and bows.
Renaud Capuçon (Franck Barley, Pf.)	1976	2010	Erato	MS	Former student of Gérard Poulet.
Alina Ibragimova (Cédric Tiberghien, Pf.)	1985	2010	Wigmore Hall Live	HIP	Former student of Valentina Korolkova, Natalya Boyarskaya, Gordan Nikolitch and Christian Tetzlaff. Previous artistic enterprises include forming the period-instrument string quartet Chiaroscuro and conducting the Academy of Ancient Music.
Midori Seiler (Jos van Immerseel, Pf.)	1969	2012	Zig-Zag Territoires	HIP	Former student of Helmut Zehetmair, Sándor Végh, and HIP specialist Thomas Hengelbrock. Previous artistic enterprises include association with the Academy for Early Music Berlin (Akamus).
Nancy Dahn (Timothy Steeves, Pf.)	1966	2013	Marquis Classics	MS	Former student of Masuko Ushioda, Glenn Dicterow and Donald Weilerstein.
Sayaka Shoji (Gianluca Cascioli, Pf.)	1983	2015	Deutsche Grammophon	MS	Former student of Zakhar Bron, Uto Ughi and Shlomo Mintz.
Pierre Fouchenneret (Romain Descharmes, Pf.)		2016	Aparte	MS	Former student of Alain Babouchian, Olivier Charlier and Devy Erlih.
Tasmin Little (Martin Roscoe, Pf.)	1965	2016	Chandos	MS	Former student at the Yehudi Menuhin School, London.
Chloë Hanslip (Danny Driver, Pf.)	1987	2017	Rubicon	MS	Former student of Natalya Boyarskaya and Zakhar Bron.
**Brahms: Violin Sonata No. 1 in G Major, Op. 78 (1879)**					
Marat Bisengaliev (Ernest Hall, Pf.)	1962	1999 (released 2010)	Universal Classics	MS	Former student of Boris Belinki and Valery Klimov.
Ilya Kaler (Alexander Peskanov, Pf.)	1963	2000	Naxos	MS	Former student of Zinaida Gilel, Yuri Yankelevich, Leonid Kogan and Viktor Tretiakov.
Nikolaj Znaider (Yefim Bronfman, Pf.)	1975	2007	Sony Classical	MS	Former student of Boris Kuschnir and Dorothy DeLay.
Anne-Sophie Mutter (Lambert Orkis, Pf.)	1963	2009	Deutsche Grammophon	MS	Former student of Erna Honigberger and Aida Stucki.
Ryoko Yano (Sergey Kuznetsov, Pf.)	1982	2009	Pan Classics	MS	Former student of Hisako Fukuzaki, Mariko Tajima, Chikashi Tanaka, Jean Jacques Kantorow and Peter Csaba.
Sihana Badivuku (Jouni Somero, Pf.)	1967	2010	FC-Records	MS	Former student of Vanco Stoilkov, Blagoja Dimcevski, Pavel Vernikov, Eugenia Chugajeva and Young Uck Kim.
Sergey Khachatryan (Lusine Khachatryan, Pf.)	1985	2012	Naive	MS	Former student of Pyotr Haykazyan, Hrachya Harutyunian and Josef Rissin.
Sunao Goko (Haruko Ueda, Pf.)	1993	2013	Ysaÿe	MS	Former student of Eshigahara Masami, Gerhard Bosse, Tatsumi Akiko, Pavel Wernikov, Jean Jacques Kantorov and Ana Chumachenko.
Kristóf Baráti (Klára Würtz, Pf.)	1979	2014	Brilliant Classics	MS	Former student of Vilmos Tátrai and Eduard Wulfson.
Christian Tetzlaff (Lars Vogt, Pf.)	1966	2015	Ondine	MS	Former student of Uwe-Martin Haiberg and Walter Levin.


Analysis of overall tempo, tempo and rhythmic alterations, multiple-stop progression, intonation, vibrato and timbre was aided by computerized software tools. Since bowings, fingerings and articulation serve as idiomatic parameters, which at present are not amenable to computerized analysis, they were obtained through meticulous repeated aural scrutiny of the relevant recordings and made by the first author (who is a professional violinist) during several listening sessions. Analysis was subsequently aided by a second violinist in order to enhance data reliability.

To reinforce the conclusions, a Fisher’s exact test was performed for the Beethoven Sonata second movement excerpt to examine the relation between style affiliation (i.e., HIP/MS) and the manner of execution of several performance attributes. Fisher’s exact test is based on exact probabilities from a specific distribution (the hypergeometric distribution) and is well suited for small sized samples. The level of statistical significance was set to alpha 0.05 in all analyses.

A fundamental aspect that was taken into account was the deficiency of most current digital waveform editors to extract information within dense polyphonic textures. In fact, one of the reasons for using Bach’s solo violin set as a source for the majority of studies was its aptness to adjust to presently available software devices: in nearly all tools, useful information could more easily be detected for small-ranged, relatively slow monophonic repertoire than for polyphonic music of varied textures.

In choosing repertoire that involves mixed instruments and thick polyphony (i.e., sonatas for violin and piano), such difficulty had to be bypassed. This was done by dividing the violin and the piano part prior to analysis using Celemony-Melodyne software DNA Direct Note Access^TM^ technology. This method enabled us to identify and edit individual notes in the polyphonic material. Melodyne is designed to single out the algorithm used for the display and playback of the various notes. The piano sound is eliminated manually, and if the same note is simultaneously played by both instruments, a single note comprising the combined sound of both instruments is automatically generated.

We subsequently applied a series of post-processing steps with the PRAAT software package version 6.0.24. PRAAT offers a wide range of standard and non-standard procedures, including dynamic, spectrographic and tempo analysis. Since data was extracted from various performances, each with its own recording amplitude, upon separating the violin from the piano, and prior to performing the measurements, we were required to multiply the amplitude of each sound in such a way that its absolute peak became the standard value (0.99). Obtaining the standard value enabled us to maximize the audibility of the sound without it being distorted. Modification of the violin part was hence made for every recording by the Scale Peak command application in such a way that its absolute peak became the new absolute peak specified for all performances. Then, each sound was labeled with TextGrid objects for annotating bars and selected notes for analysis.

An urtext edition of the examined repertoire (as published by G. Henle Publishers) was used in all cases as a reference point (Brandenburg, 1978).

## Results

### Dynamics

**FIGURE 1:**
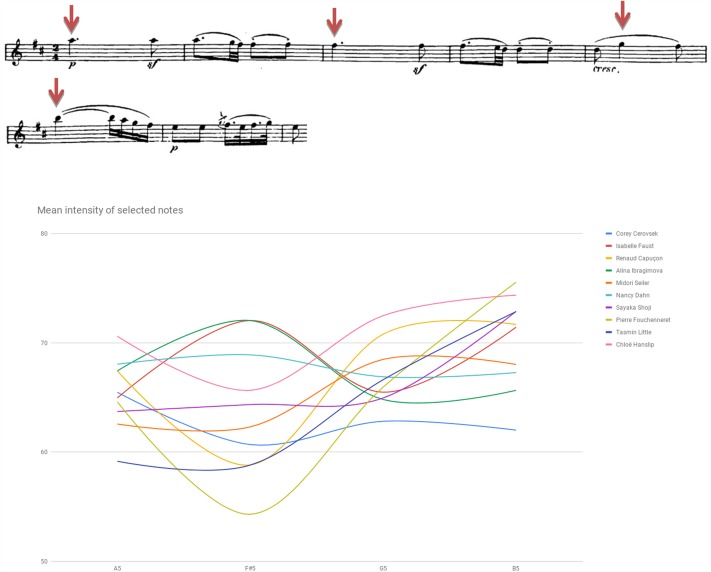
Mean intensity of selected notes (dynamic contour) in dB SPL: Beethoven Violin Sonata No. 6 in A Major Op. 30 No. 1, second movement (Adagio molto espressivo), bars 1–6.

**FIGURE 2:**
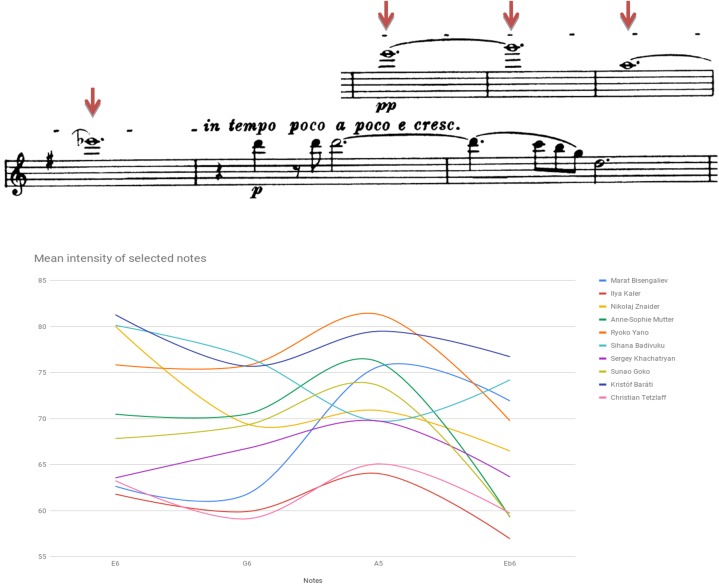
Mean intensity of selected notes (dynamic contour) in dB SPL: Brahms Sonata first movement (Vivace man non troppo), bars 219–224.

Figure 1 plots mean intensity contour (i.e., dynamics spectrum) as traced in selected notes of Beethoven Sonata’s second Movement, bars 1–6. Figure 2 plots mean intensity contour as traced in selected notes of Brahms Sonata’s first movement, bars 219–224 (affixed is a score of the segment).^3^ Noted for the Beethoven renderings are Cerovsek and Ibragimova’s soft, more silent playing as opposed to Hanslip and Dahn’s high-powered reading. In the Brahms readings noted are Znaider’s whispered, subdued execution or Kaler’s soft-toned portrayal, both at odds with the general inclination toward a more strident rhetoric.

An analysis of selected notes from Beethoven Sonata’s second movement excerpt was conducted for the use of Messa di Voce. Two of the three HIP performers (66.7%) and four of the seven MS players (57.1%) have notably implemented the device, a difference that is not statistically significant (*p* = 0.667, Fisher’s exact test).^4^

An examination of Beethoven Sonata’s first movement indicates several noted effects in dynamics, such as the use of Messa di Voce to enhance tension, exhibited by Ibragimova (e.g., E4 bars 38–40, D6 bar 124), Seiler (e.g., E4 bars 38–40, F#4 bar 65, D#4 bar 66), and Hanslip (e.g., bars 38–40, 120, 124), or the use of terraced dynamics for inner-phrase deviation employed by Fouchenneret (e.g., second repeat bars 69–75, 229–232) or Faust (e.g., bars 76–79, 235–237).

In Brahms Sonata’s first movement, Messa di Voce was displayed by Badivuku (e.g., B2 bar 11, D#3 bar 12, B2 bar 14, double-stops 29–35). Also noted were the frequent changes of dynamics (assisted by alterations of tempo, bow pressure and vibrato) used for phrase reiterations (e.g., Tetzlaff, bars 3–5, 5–7, Mutter and Khachatryan, bars 11–13), or to enforce phrase rhetoric by displaying note emphasis, sudden volume shifts or dynamics reiteration (e.g., Badivuku, bars 48–50; Khachatryan, bar 1; Goko, bars 6–8, Baráti diminution of A4, bar 18).

Table 3 summarizes the main findings in regards dynamics. All in all, diverse dynamics spectrum was traced, used as an aid for phrase shaping and contour and affected by the musical context. The use of Messa di Voce or the manifestation of terraced dynamics were exhibited regardless of performer’s age, recording date, style affiliation or schooling.

**Table 3:** Dynamics: summary of the main findings.

	Soft, tender dynamic spectrum	High powered dynamic spectrum	Use of Messa di Voce	Use of terraced dynamics	Frequent inner-phrase dynamics changes
Performer	Cerovsek, Ibragimova^∗^, Znaider, Kaler	Hanslip, Dahn	Ibragimova^∗^, Seiler^∗^, Faust^∗^, Shoji, Cerovsek, Little, Hanslip, Badivuku	Fouchenneret, Faust^∗^	Tetzlaff, Mutter, Khachatryan, Badivuku, Goko, Baráti


*^∗^HIP violinist.*

### Overall Tempo

Table 4 displays mean tempo of the analyzed movements of both Beethoven and Brahms sonatas. No clear-cut trends were evident with regards to tempo preferences: Fouchenneret exhibits the slowest tempo in Beethoven’s first movement, yet his rendering of the second movement alludes to close-to-average velocity. Similarly, Shoji’s adaptation of a rather standard tempo in the Allegro does not attest to the relatively slow pace chosen for the following movement. Exceptional is Dahn’s clear preference for extreme, polar paces, manifested in her utilization of the fastest and slowest tempi (respectively) for both movements among the performers examined. Also noted is the wide range of tempo choices, most evident in the playing of Beethoven’s first movement but also recognizable in the various renderings made of the Brahms.

**Table 4:** Mean tempo of the analyzed movements.

Performer	Beethoven Violin Sonata		Performer	Brahms Violin Sonata
			
	Mov. 1 (Allegro): Mean BPM	Mov. 2 (Adagio molto espressivo): Mean BPM		Mov. 1 (Vivace ma non troppo): Mean BPM
Corey Cerovsek	99	61	Marat Bisengaliev	138
Isabelle Faust	114	62	Ilya Kaler	138
Renaud Capuçon	101	65	Nikolaj Znaider	143
Alina Ibragimova	114	58	Anne-Sophie Mutter	131
Midori Seiler	102	61	Ryoko Yano	132
Nancy Dahn	140	52	Sihana Badivuku	127
Sayaka Shoji	100	56	Sergey Khachatryan	130
Pierre Fouchenneret	93	58	Sunao Goko	138
Tasmin Little	100	59	Kristóf Barti	145
Chloë Hanslip	104	63	Christian Tetzlaff	139

An examination of the relation between style affiliation (i.e., HIP/MS) and the average tempo exhibited in Beethoven’s Sonata second movement showed lower than average tempo among two of the three HIP players (66.7%) and three of the seven MS performers (42.9%), a difference that is not statistically significant (*p* = 0.5, Fisher’s exact test).^5^

Altogether, a wide range of tempo preferences was observed. No clear connection could be found between presumed association to either HIP or MS and the tempo chosen for performance. As reported in previous studies, tempo choices are dependent on personal inclinations rather than connected to background factors such as age, recording date, schooling or style affiliation.

### Tempo and Rhythmic Alterations

**FIGURE 3:**
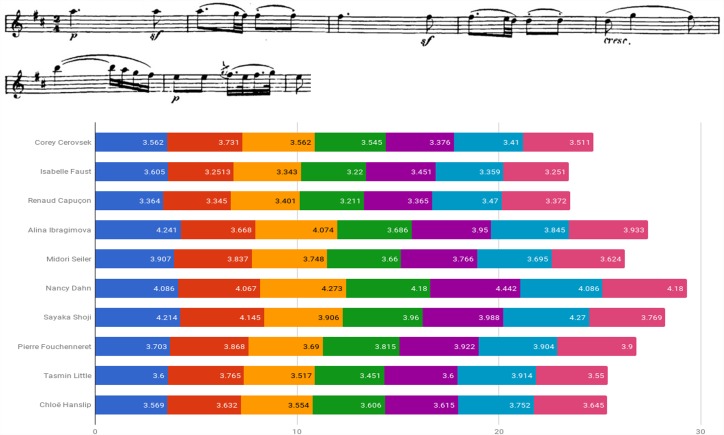
Bar to bar duration (in seconds): Beethoven Violin Sonata No. 6 in A Major Op. 30 No. 1, second movement (Adagio molto espressivo), bars 1–6.

Figure 3 plots inner phrase modifications found in bars 1–6 of Beethoven Sonata’s second movement by calculating durations of each of the bars (affixed is a score of the segment). Amidst extensive variety, there is a salient inclination toward a wide range of accelerando and ritardando presented by HIP violinists Ibragimova and Faust, as opposed to the relatively stable pace traced in the playing of Cerovsek, Seiler and Fouchenneret. Also discernible is the overall slowing down by Cerovsek and Seiler, versus the gradual increase in tempo displayed by Dahn and Shoji.

An examination of the relation between style affiliation (i.e., HIP/MS) and the tempo modifications exhibited in the Beethoven excerpt showed notable use of the device among two of the three HIP players (66.7%) and three of the seven MS performers (42.9%), a difference that is not statistically significant (*p* = 0.5, Fisher’s exact test).^6^

Compared to the second movement’s findings, examining the Sonata’s first movement (Allegro) points to rather different choices made by the same performers, indicating that established individual trends are far from being set in stone. Opposite in characteristics to the second movement’s results are the tempo modifications exhibited by Seiler (e.g., bars 60, 64, 213, 217) or Fouchenneret’s long ritardando during section endings (e.g., bars 148–150, 248–249).

**FIGURE 4:**
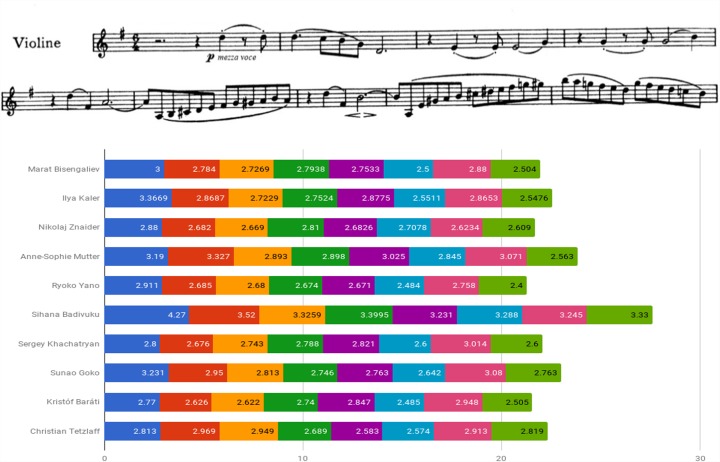
Bar to bar duration (in seconds): Beethoven Violin Sonata No. 1 in G Major Op. 78, first movement (Vivace man non troppo), bars 1–8.

Examining Brahms Sonata’s first movement, a similar tendency toward tempo alterations emerges: Figure 4 plots inner phrase modifications analyzed for bars 1–8 by calculating the duration of each of the bars (affixed is a score of the segment). While considerable alterations were exhibited by all violinists, particularly salient is the tendency for extensive inner-phrase tempo changes manifested by Bisengaliev, Khachatryan and Baráti, and the use of a relatively stable pace by Badivuku. Bisengaliev’s approach is found as a trend characteristic (e.g., bars 10, 15, 19–20, 23–25, 43, 55–59), and Mutter was also observed as using extreme rubato for outlining inner phrases and to differ between repeated sequences (e.g., bars 11–13, 23–29, 34–35, 43, 50). Similar trends were found in Tetzlaff’s rendering, as he employs inner phrase rubato (e.g., bars 11–13, 28–29, 46, 57) coupled with rapid dynamic changes used for color and shadings. Kaler and Znaider exhibit accelerandos and ritardandos upon long musical lines.

**FIGURE 5:**
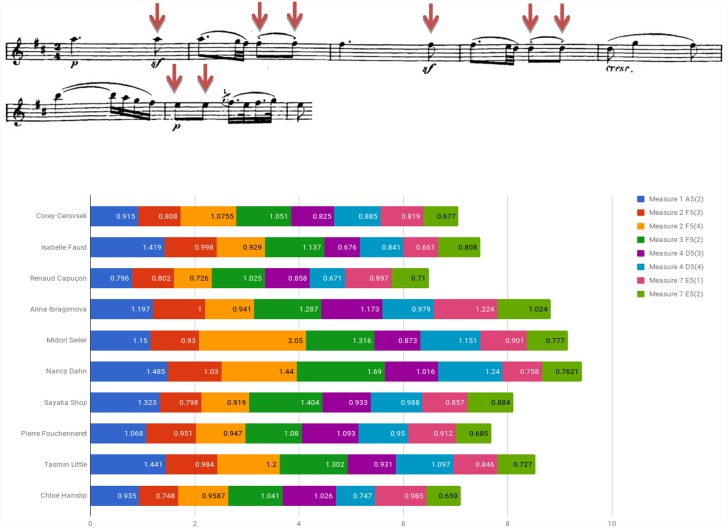
Eighth note durations (in seconds): Beethoven Violin Sonata No. 6 in A Major Op. 30 No. 1, second movement (Adagio molto espressivo), bars 1–6.

Figure 5 examines the use of agogic accents in bars 1–6 of Beethoven Sonata’s second movement by presenting the length of both individual and consecutive eighth notes of the segment (indicated by downward red arrows affixed to the score). Consecutive eighth notes were found in various lengths, a pair’s subsequent note either lengthened (e.g., Dahn, Shoji), shortened (e.g., Capuçon, Ibragimova, Fouchenneret) or varied in measure (e.g., Cerovsek, Little, Hanslip).

An examination of the relation between style affiliation (i.e., HIP/MS) and the use of agogic accents exhibited in the Beethoven excerpt showed notable use of the device among two of the three HIP players (66.7%) and two of the seven MS performers (28.6%), a difference that is not statistically significant (*p* = 0.333, Fisher’s exact test).^7^

Throughout the Sonata’s first movement, lengthening or shortening of half, quarter or eight notes within various rhythmic figures were exhibited by several violinists, such as Cerovsek (e.g., bars 61, 75, 131), Faust (e.g., bars 67, 106, 138–139), Dahn (e.g., bars 76–79, 229–232), Little (e.g., bars 54, 138–139), Capuçon (e.g., bars 54, 122, 126) or Ibragimova (e.g., bar 138–139), the latter exhibiting a general inclination toward the shortening of notes.

**FIGURE 6:**
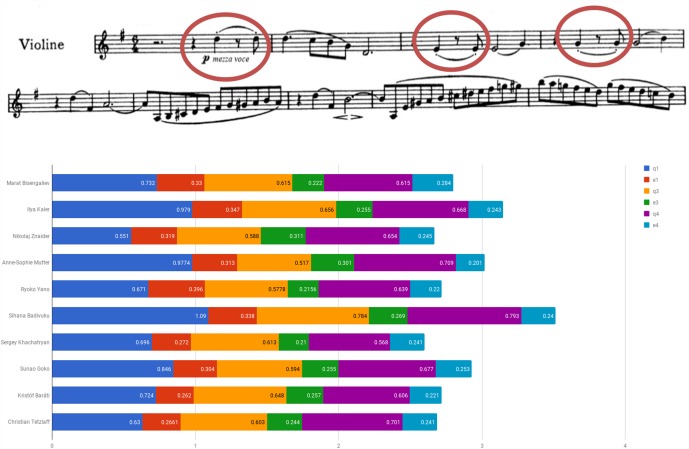
Quarter and eighth note duration of repeated rhythmic figures (in seconds): Beethoven Violin Sonata No. 1 in G Major Op. 78, first movement (Vivace man non troppo), bars 1–8.

Figure 6 examines the use of agogic accents in the Brahms Sonata by presenting the length of the repeated figure quarter – eighth note stop – eighth note, originally marked with a dash (indicated by circles affixed to the score). One can detect Znaider’s penchant for lengthening the eighth notes, Badivuku’s inclination for their far-reaching reduction, the relative preservation of the noted ratio displayed by Yano and Goko, and the change in rhythmic values of quarter and eighth notes upon the figure reiterations exhibited by the majority of players. Throughout the movement one could trace an abundant usage of the device, such as in the playing of Bisengaliev (e.g., bars 22, 29–34), Badivuku (e.g., bars 23–24, double-stops bars 33–35), Khachatryan (e.g., bars 23, 70–71, 74) or Tetzlaff (e.g., bars 9, 17, 20, 33). Several players retain individual trends, such as Badivuku’s inclination toward shortening of notes, Yano’s lengthening of double-stops, or Baráti’s shortening of the last eighth note in the figure quarter – dotted quarter note – eighth note (e.g., bars 40–41, 43), and in dotted figures (e.g., bar 48).

Melodic rubato was traced by only one violinist (i.e., Bisengaliev, bars 21, 40–44), implying that such rhythmic feature still fails to pass present-day conventions.

Table 5 summarizes the main findings in regards tempo and rhythmic alterations. All in all, modifications were traced among many of the examined violinists in various degrees and in diverse locations, regardless of personal background or style affiliation, thus corresponding to both HIP and 19th century performance traditions which champion frequent tempo and rhythmic alterations. Tempo fluctuations and agogic accents seem to be connected to musical texture rather than to personal idiosyncrasies, although several players have certainly displayed individual trends.

**Table 5:** Tempo and rhythmic alterations: summary of the main findings.

	Considerable use of inner phrase modifications (accelerando–rallentando)	Utilization of a relatively stable pace	Gradual accelerando∖ rallentando upon long segments	Displaying contrasting tempo characteristics in between the various movements	Considerable use of agogic accents	General preservation of the noted ratio	Utilization of melodic rubato
Performer	Ibragimova^∗^, Faust^∗^, Dahn, Little, Bisengaliev, Khachatryan, Baráti, Mutter, Tetzlaff	Cerovsek, Badivuku	Cerovsek, Seiler^∗^, Dahn, Shoji, Kaler, Znaider	Seiler^∗^, Fouchenneret	Cerovsek, Faust^∗^, Seiler^∗^, Dahn, Little, Capuçon, Ibragimova^∗^, Znaider, Badivuku, Bisengaliev, Khachatryan, Tetzlaff	Yano, Goko	Bisengaliev


*^∗^HIP violinist.*

That said, overall listening to the recordings at hand reveals that albeit considerable variety of tempo and rhythmic manifestations, an underlying metric level is maintained throughout long passages, providing a sense of systematical groupings and producing a feeling of temporal regularity regardless of local durational shifts. As opposed to the eccentric, capricious and rather extemporary manifestation of tempo and rhythm prevalent in 19th century praxis, present-day violinists portray a sense of temporal stability and large-scale, “architectural” phrase formation. Noticeable in this regard are Shoji, Kaler and Znaider, who seem to exhibit accelerandos and ritardandos upon long musical strains, providing a sense of systematization and intentional strategic ordering. Similarly, Goko and Yano remain quite loyal to the notated rhythmic values. In this sense, these violinists appear to depart from 19th century praxis with regards to rhythmic manifestations and seem to be much more restrained in their interpretation than the trends implied in the early recording decades.

### Multiple-Stop Progression

**FIGURE 7:**
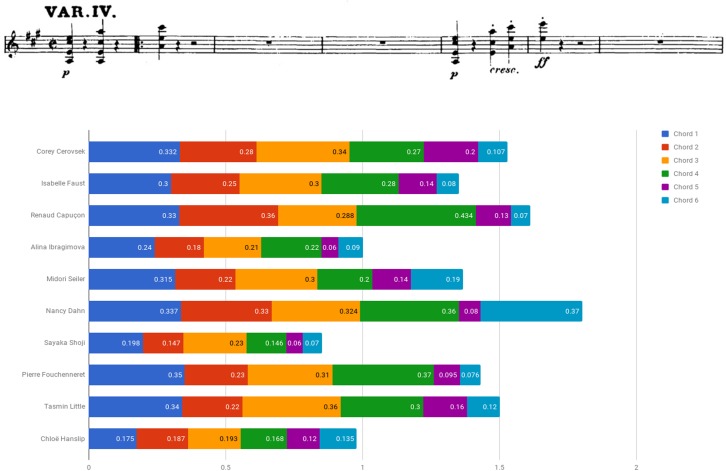
Multiple-stops durations (in seconds): Beethoven Violin Sonata No. 6 in A Major Op. 30 No. 1, third movement (Allegretto con variazioni – variation IV), bars 81–85.

**FIGURE 8:**
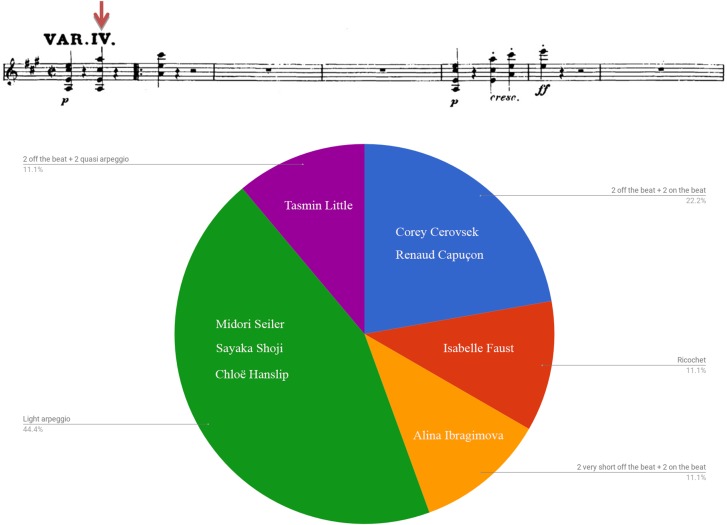
Chord manner of execution: Beethoven Violin Sonata No. 6 in A Major Op. 30 No. 1, third movement (Allegretto con variazioni – variation IV), bars 81, beat 3 (AM).

Figure 7 plots duration of multiple-stops found in bars 81–85 of Beethoven Sonata’s third movement (Allegretto con variazioni – Variation IV) by calculating the ratio between the lowest and the highest notes of the sampled chords (affixed is a score of the segment). Figure 8 exhibits the various manners of execution of one of the chords (indicated by a downward red arrow affixed to the score). It is evident that there was a wide variety of means by which the multiple-stops were executed: Dahn displayed a relatively equal execution of the multiple-stops rhythmic value, while most others demonstrated a wide diversity in both length and manner of execution of the various chords, including arpeggio, ricochet and diverse divisions.^8^

An examination of the relation between style affiliation (i.e., HIP/MS) and multiple-stop realization exhibited throughout the excerpt showed notable use of arpeggio and ricochet among two of the three HIP players (66.7%) and four of the seven MS performers (57.1%), a difference that is not statistically significant (*p* = 0.667, Fisher’s exact test).^9^

All in all, findings indicate the diversity of multiple-stop renderings, regardless of performers’ background. No connection was found between performers’ style affiliation and the manner of execution of chords: both HIP and MS performers tended to choose arpeggiation or ricochet as well as various other interpretations.

### Intonation Profile

**FIGURE 9:**
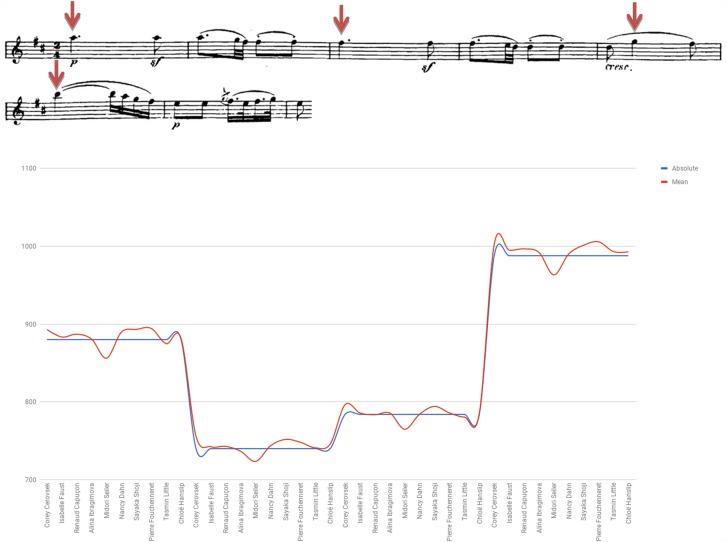
Deviation from absolute pitch: Beethoven Violin Sonata No. 6 in A Major Op. 30 No. 1, second movement (Adagio molto espressivo), A5 bar 1, F#5 bar 3, G5 bar 5, B5 bar 6.

Figure 9 presents an intonation profile found in bars 1–6 of Beethoven Sonata’s second movement, by showing the pitch deviation between the measured mean F0 of a few selected notes (indicated by downward red arrows affixed to the score) and their F0 value in equal temperament (the latter used as reference intonation with a standard tuning of 440 Hz). Noted are Seiler’s use of a relatively low pitch, and the persistence of intonation tendencies featured by the majority of players, save (to a minor extent) Dahn, Capuçon, and Fouchenneret, who exhibited the notes in various distances from their mean pitch value.

An analysis of selected notes from the Beethoven excerpt was conducted for the use of lower deviation from the absolute frequency value (*A* = 440). One out of the three HIP performers (33.3%) and none of the MS players (0%) have exhibited lower pitch than absolute, a difference that is statistically insignificant (*p* = 0.3, Fisher’s exact test).^10^

**FIGURE 10:**
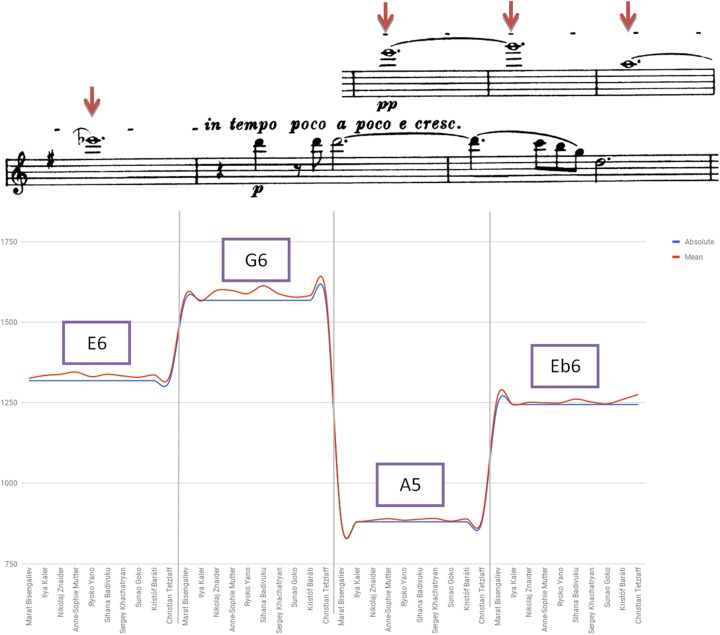
Deviation from absolute pitch: Brahms Violin Sonata No. 1 in G Major Op. 78, first movement (Vivace man non troppo), E6 bar 219, G6 bar 220, A5 bar 221, Eb6 bar 222.

Figure 10 presents findings detected in bars 219–222 of the Brahms Sonata’s first movement. While no performer used a pitch that was lower than the standard absolute, a slight tendency for a higher pitch level was found in the playing of Tetzlaff and Badivuku, while Kaler, Yano and Goko displayed a rather steadfast intonation conforming to absolute standard tuning. Several violinists tended to raise the first two notes, noticeable for their high range, upon lowering the Eb6 of bar 222 due to its function as a leading-note to the following D Major tonality (e.g., Mutter, Znaider, Badivuku).

Table 6 summarizes the main findings in regards intonation profile. On the whole, it seems that steadfast intonation, regardless of pitch or temperament model, tends to dominate performers’ habitual practice. Variations in pitch level were certainly found, yet consistency of intonation profile and compliance to standard tuning seems most widespread, blurring divergence and idiosyncrasies. Differences between HIP and MS performers in regards utilization of low pitch tuning was found to be statistically insignificant, questioning the assumption as to its higher usage among pre-1800 specialists who perform 19th century music.

**Table 6:** Intonation profile: summary of the main findings.

	Use of a low pitch	Noted divergence from mean pitch value	Tendency for a higher pitch level than the standard absolute	Intonation conforming to absolute standard tuning
Performer	Seiler^∗^	Dahn, Capuçon, Fouchenneret	Tetzlaff, Badivuku	Cerovsek, Faust^∗^, Ibragimova^∗^, Shoji, Little, Hanslip, Bisengaliev, Kaler, Znaider, Mutter, Khachatryan, Yano, Goko, Baráti


*^∗^HIP violinist.*

### Vibrato

**FIGURE 11:**
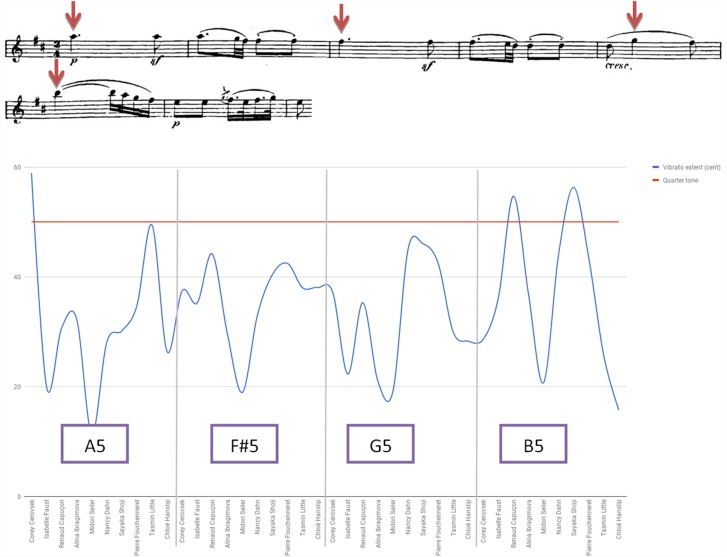
Vibrato extent (in cents): Beethoven Violin Sonata No. 6 in A Major Op. 30 No. 1, second movement (Adagio molto espressivo), A5 bar 1, F#5 bar 3, G5 bar 5, B5 bar 6.

Figure 11 plots vibrato extent (i.e., depth) found in selected notes of bars 1–6 of Beethoven Sonata’s second movement, calculated for the average of a note’s pitch contours (i.e., its peaks and valleys) compared to its mean pitch undulations.^11^ An analysis was also conducted for vibrato rate (i.e., speed) and onset.

Noted were Seiler’s use of a wide pitch and low speed vibrato, and Faust’s late onset of vibrato after a note’s starting point, both violinists associated with HIP. Also noticeable was the deviation found regarding vibrato depth within Capuçon’s playing, as opposed to the relative consistency in this regard in Fouchenneret’s playing, the fast vibrato featured by Shoji, the consistency of speed found in Dahn’s playing, the early vibrato onset presented by Ibragimova, and the variety of vibrato onset exhibited by Hanslip.

An analysis of selected notes from the Beethoven excerpt was conducted for the use of narrow vibrato. Three of the three HIP performers (100%) and two of the seven MS players (28.6%) exhibited lower pitch than absolute, a difference that approached near significance (*p* = 0.083, Fisher’s exact test) and might attest to the higher degree of its usage among HIP players while playing 19th century music.^12^

Examining the Sonata’s first movement, sparse usage and a rather shallow vibrato was found in the playing of MS representatives such as Capuçon, Dahn, Shoji, Little or Hanslip, as well as in the recordings of HIP violinists Faust, Seiler and Ibragimova. On the opposite side, Fouchenneret exhibits what could be regarded as a more orthodox performance style, displaying a rather wide and continuous vibrato.

**FIGURE 12:**
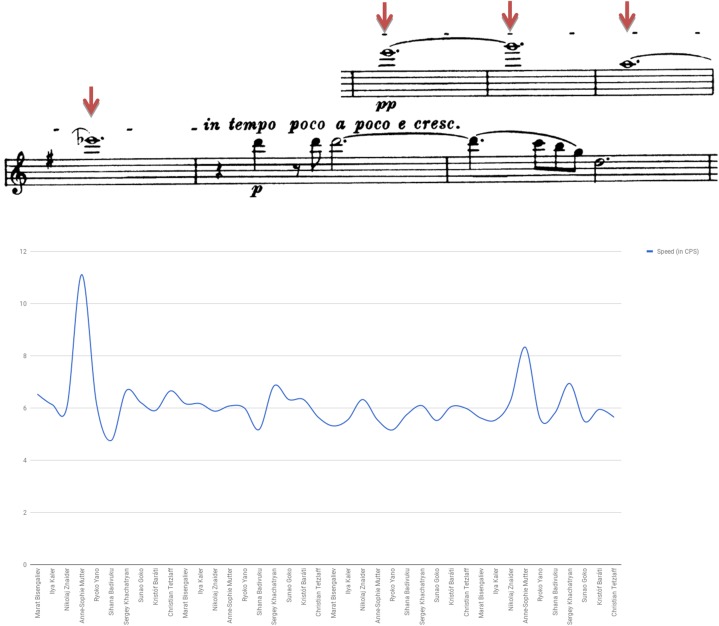
Vibrato speed (in CPS): Brahms Violin Sonata No. 1 in G Major Op. 78, first movement (Vivace man non troppo), E6 bar 219, G6 bar 220, A5 bar 221, Eb6 bar 222.

Figure 12 presents vibrato rate (i.e., speed) detected in bars 219–222 of Brahms Sonata’s first movement. Analysis was additionally made for vibrato extent and onset. Notable was the use of narrow vibrato by Bisengaliev and Yano, versus the relatively wide vibrato featured by Znaider. Also notable were the differences of vibrato extent exhibited for the various notes, most conspicuous in the playing of Mutter and Baráti. Mutter uses the fastest vibrato, while most violinists tend to oscillate in the relatively slow pace of 5–6, thus complying with the phrase’s somewhat ambiguous character. On the whole, it seems that Kaler exhibits the latest vibrato onset and Khachatryan the earliest, yet this feature surely varies between the notes among all players. Noted, for example, was Tetzlaff’s extreme delay in vibrating the phrase’s first note as opposed to his quite early oscillation later on, or Mutter’s late onset displayed in the segment’s second note as opposed to its early feature in the note preceding.

Diverse manners of vibrato execution with regards to width, speed, onset, and duration were found throughout the Brahms movement: Znaider employs sparse usage of vibrato in several passages, including omitting it altogether during highly charged locations (e.g., F#4, bar 60). Mutter varies her vibrato with regards to width, speed, onset, and duration, using slow and wide vibrato during dark and more contemplative figures (e.g., bars 1–5, 11–18), narrow and fast during dramatic events (e.g., bars 23–29, 40–44), and non-vibrato during low-registered, ‘dim’ fragments (e.g., bars 58–69). Badivuku uses different manners of vibrato for the molding of specific phrases and for shaping highly expressive contours: she employs wide and slow vibrato during soft, ‘airy’ passages (e.g., bars 36–40, 61–66), fast and narrow vibrato during dramatic, high-registered phrases or in order to enforce dynamics elevation (e.g., bars 25–29, 48–50, 51–53), scant, late onset and at times even non-vibrato during notes or figures of exalted emotional content (e.g., bars 1–3, 67–68, bar 67, 68, 78–81). Khachatryan presents a narrow, soft, and very gentle vibrato, almost omitted at times (e.g., bars 66–70). Baráti resorts to a stable and fast vibrato, yet in several passages implements late onset (F#5 bar 53, A3 bar 67), sparse (e.g., bars 78–81) and even non-vibrato (e.g., B5 bar 52, bars 63–64).

Table 7 summarizes the main findings in regards vibrato. Overall, vibrato was implemented in various manners, including long fragments of sparse usage or even total lack of the device, and regardless of biographical background or school. The different manners of vibrato execution contributed to the molding of specific phrases and were shaped according to the musical and emotional contour, by thus obfuscating individual idiosyncrasies and stylistic trends. Vibrato has surely been displayed in various modes throughout all periods, yet the manner and degree of its scarce usage suggests inclination toward assumed 19th century performance conventions as well as HIP style influences. While an established trend for the use of narrow vibrato was traced among HIP players more so than among the MS peers, the limited use of this device, being equally evinced by both HIP and MS players, invalidates the assumption as to its more extensive implementation among HIP violinists.

**Table 7:** Vibrato: summary of the main findings.

	Noted use of wide/narrow pitch	Noted use of low/high speed	Noted display of early/late onset	Varied vibrato depth within the note/excerpt	Varied vibrato speed within the note/excerpt	Varied vibrato onset within the note/excerpt	Sparse usage of vibrato
Performer	Seiler^∗^, Faust^∗^, Ibragimova^∗^, Dahn, Fouchenneret, Bisengaliev, Yano, Znaider	Seiler^∗^, Shoji, Mutter	Faust^∗^, Ibragimova^∗^, Baráti	Capuçon, Mutter, Badivuku	Mutter, Badivuku	Hanslip, Kaler, Khachatryan, Tetzlaff, Mutter, Badivuku	Capuçon, Dahn, Shoji, Little, Hanslip, Faust^∗^, Seiler^∗^, Ibragimova^∗^, Mutter, Baráti, Znaider, Khachatryan


*^∗^HIP violinist.*

### Timbre

**FIGURE 13:**
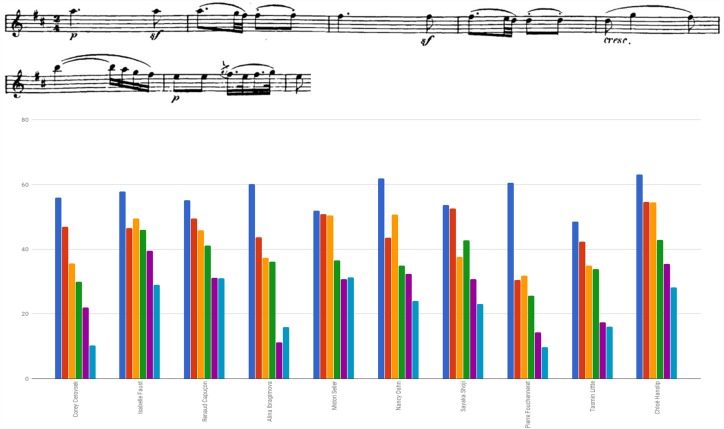
Spectral components: Beethoven Violin Sonata No. 6 in A Major Op. 30 No. 1, second movement (Adagio molto espressivo), A5 bar 1.

Timbre shadings, generated through different means of bowing (e.g., bow position, pressure, acceleration, bow tilt, bow-bridge distance), serve as a substantial device in the molding of notes and phrases. Figure 13 displays the spectral components (i.e., the note’s fundamental frequency and first six harmonics) found for Beethoven Sonata’s second movement’s very first note (A5, bar 1 beat 1) examined for their sound pressure level in Hertz (dB/Hz). Noted are Seiler’s and Shoji’s inclination toward a ‘bright,’ lighter sound versus the ‘darker,’ richer coloring exhibited by Cerovsek, Ibragimova and Fouchenneret. Examining the Sonata’s first movement, a general penchant for a somewhat tender, soft sound was observed as a tool used for phrase reiterations or to differentiate between repeated notes (e.g., Ibragimova’s use of soft, light, and ‘airy’ shadings, bars 10–19, 69–75, 142–148, 222–227, Fouchenneret, Shoji or Hanslip’s somewhat ethereal timbre shadings, bars 61–63, 69–75, 214–215, 222–227). Seiler’s recording includes the use of a period violin combined with gut strings and period bow, affecting timbre as well as the overall execution of related performance parameters.

**FIGURE 14:**
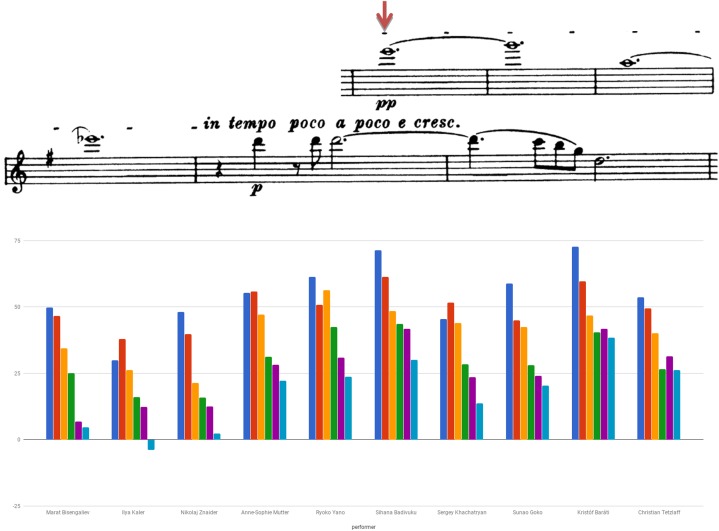
Spectral components: Brahms Violin Sonata No. 1 in G Major Op. 78, first movement (Vivace man non troppo), A5 bar 222.

Figure 14 displays the spectral components found for A5, bar 221 of Brahms Sonata’s first movement, examined for their sound pressure level in Hertz (dB/Hz). Noted are Khachatryan’s and Kaler’s inclination toward ‘bright,’ somewhat ‘airy’ sound as opposed to the ‘darker,’ thick coloring exhibited by Baráti and Goko. Throughout the movement, Znaider, Mutter and Tetzlaff use sul tasto/flautando aided by playing in the upper half of the bow to display soft, ‘airy’ colorings (e.g., bars 1–10, 54–60, 61–66), differentiating between phrases by using various amount of pressure and bow placement.

Altogether, various means of shadings were depicted, and there seems to be no relevance to school or style-presumed affiliation with regards to timbre contour. As exhibited in further features, it was associated with phrase characterization and textual outline. Although extensive scrutiny is still needed for deciphering performers’ distinguishable tone-color peculiarities, the general employ of diverse hue can be seen not only as an influence of late 20th century praxis, but also as a manifestation of individuality and personal imprint.

### Bowings and Fingerings

Repeated aural scrutiny of the Beethoven Sonata has found a tendency toward using first position, open strings and un-noticeable position shifts in the playing of both MS (e.g., Little, Hanslip) and HIP (e.g., Faust, Ibragimova) performers. HIP players were also found to add harmonics, used for the delineation of gentle, soft passages (e.g., bars 122, 146–147, 214, 249). At the other pole, Capuçon avoids the use of open strings by fingering the note on its adjacent lower string. Audible position shifts and the use of portamento was exhibited by Cerovsek, Dahn, Shoji, Fouchenneret and Seiler. Several changes made to the original notated bowings were traced in the playing of Cerovsek (e.g., bars 119–130), Capuçon (e.g., bars 10–15, 26, 70, 109–110, 113–114), Dahn (e.g., bars 76, 112–113, 198, 229), Shoji (bars 10–15, 25, 82, 165, 183–184) and Little (e.g., 183–184, 229–230, 233), carried out for both technical and contextual purposes. HIP players were generally observant of the urtext score, despite minor bowing changes (e.g., Faust- bars 27, 58 second repeat, 125–126, Seiler’s added slurs at bar 72–74).

As for the Brahms Sonata, extensive use of portamento and audible position shifts was traced in the playing of Bisengaliev, Znaider, Badiuku and Goko (e.g., bars 5, 7, 11, 15, 16, 18–19, 37, 39, 44, 49, 58, 112), and made to enforce phrase division (e.g., Kaler’s use of first position in bar 50 for a brighter, tenser sound, or his position shift in F5, middle of bar 54 used for a more delicate sound). Yano and Khachatryan appear at the opposite pole with their inclination toward inaudible position shifts and rare portamento. Open strings were used sporadically by the majority of players, while harmonics were employed to a minor scale in specific spots (e.g., bars 5, 58), and traced in the playing of Kaler, Znaider, Badivuku Mutter and Baráti. Bow changes varying from the original score were exhibited by the majority of players, most noticeable of which were Kaler (e.g., bars 17–18, 59–60, 68–69, 70–71, 107), Znaider (e.g., bars 34–35, 51, 98) and Yano (e.g., bars 19–20, 28, 54–59, 93). More observant of the initial script, although still displaying minor changes, were Mutter, Goko, Baráti, and Tetzlaff.

Table 8 summarizes the main findings in regards bowings and fingerings. On the whole, findings point to a distinction made between early and late 19th century repertoire, the former interpreted as more suitable to HIP practices in its wider use of first position, open strings and un-noticeable position shifts, whereas the latter as concurrent to 19th century traditions through its wider exhibition of portamento and audible position shifts. The general tendency toward bowing modifications, traced more among MS players than among their HIP peers, corresponds to the predominance of HIP agenda among the latter. Distinctive peculiarities and individual trends shown to affect performer’s choices, such as Little and Yano’s avoidance of audible shifts, or Kaler’s position choices made to enforce musical phrasing.

**Table 8:** Bowings and fingerings: summary of the main findings.

	Inclination toward the first position	Considerable use of open strings	Unnoticeable position shifts	Use of portamento	Implementation of harmonics	Changes made to the original notated bowings
Performer	Little, Hanslip, Faust^∗^, Ibragimova^∗^	Little, Hanslip, Faust^∗^, Ibragimova^∗^	Little, Hanslip, Faust^∗^, Ibragimova^∗^, Yano, Khachatryan	Bisengaliev, Znaider, Badiuku, Goko, Kaler	Faust^∗^, Ibragimova^∗^, Seiler^∗^, Kaler, Znaider, Badivuku Mutter, Baráti	Cerovsek, Capuçon, Dahn, Shoji, Little, Kaler, Znaider, Yano


*^∗^HIP violinist.*

### Articulation

Various means of articulation and bowing effects were used for notes or sections of special significance. Examining Beethoven Sonata’s first movement, one could trace the employment of articulation devices as a special effect (e.g., Faust’s use of flautando in bars 146–147, Dahn’s accentuated B4 of bar 129, Hanslip’s use of ponticello in bars 69–72). Seiler exercised a wide spectrum of articulation, including the use of détaché (i.e., 16th notes of bars 56–59, 116–118, 130, 208–212) and accentuated strokes (e.g., bars 69, 88–93, 138, 184–185). Notes originally marked with a dash were either observed through a bouncing, spiccato stroke (e.g., Cerovsek, bars 59–60, Hanslip, bars 60, 63–64, 213) or by exhibiting long, tenuto strokes (e.g., Shoji and Fouchenneret, bars 54, 60, 207–208). Yet generally speaking, most renderings of the movement have leaned toward a somewhat ‘clean,’ unaltered oeuvre.

Examining the Brahms Sonata, a more varied picture emerges: portato was used in order to enforce dramatic progression (e.g., Bisengaliev, bars 25–26; Khachatryan, bars 8–10, 21, 36–44). Badivuku changed articulation to enforce differences between congruous sequences (e.g., using light, bouncy strokes in bars 70–71 as opposed to detached strokes in bars 74–75) and varied bowing technique for specially punctuated notes (e.g., bouncing the dashed quarter notes E5, C#5, B4 of bar 71). Goko applied a dance-like character to bars 70–71 by separating the notated slurs while using short, light bows. Baráti stressed down-bows during the setting off of long notes (e.g., A4 bar 5, B3 bar 11, D#4 bar 12, D4 bar 13, A4 bar 45), and generally punctuated the notes to produce shine and sparkle. Tetzlaff resorted to an overall soft, tender color manifested by using the upper, lighter part of the bow and through the use of flautando (e.g., bars 61–66).

Idiosyncrasies and distinct tendencies were again spotted, such as Cerovsek’s ‘clean’ rendering exhibited by rather smooth, long bowings or Mutter’s awareness for a manifold of sound colors achieved via the use of brisk and energetic punctuation during conspicuous, intensive spots (e.g., bars 40–43, 49), as opposed to portraying soothed sounds during phrases of a less strenuous and gentler nature (e.g., bars 1–10, 54–60).

Table 9 summarizes the main findings in regards articulation. All in all, modest usage of distinct articulation devices was found among the Beethoven Sonata performers. This could be attributed to its apprehension as a ‘Classical’ work, hence retaining a rather serene and formal character. Seiler’s employment of a wide palette of articulation features could thus be considered as more in line with Baroque practices than with the period herewith. On the other hand, extensive utilization of articulation devices was found in the Brahms Sonata recordings. This not only attests to the overall expansion of performance options and idiomatic range, but also somewhat complies with period conventions: Exhibiting light and bouncy strokes as a means to bestow a whimsy and capricious quality to the music, is considered a well-established feature of late 19th century praxis. Although still far from the improvisational nature of the Joachim-Berlin school, present-day performers seem to come closer to its peculiarities with regards to several articulation characteristics.

**Table 9:** Articulation: summary of the main findings.

	Implementation of détaché/tenuto strokes	Portato strokes	Light, bouncing strokes	Punctuated, stressed notes	Light, ‘airy’ bow strokes
Performer	Seiler^∗^, Shoji, Fouchenneret, Badivuku, Cerovsek	Bisengaliev, Khachatryan,	Cerovsek, Hanslip Badivuku, Goko	Seiler^∗^, Baráti, Dahn, Mutter	Seiler^∗^, Faust^∗^, Ibragimova^∗^, Hanslip Tetzlaff, Mutter


*^∗^HIP violinist.*

## Discussion

Prior to conclusion, one should acknowledge the limitation of our study in reaching comprehensive, steadfast deductions. The small amount of HIP players examined requires that far-reaching statements be taken with caution. So is the relatively limited data used for investigation, based on excerpts from two musical pieces and their renderings by a moderate group of performers. Bearing in mind the limited scope of our research we also decided not to fully indulge in core constituents of two of the analyzed performance features: portamento was observed without regard to duration, range, direction, intensity or fingerings, and no analysis was made as to bowings direction and placing.

Observing the main insights gained from the data, most palpable is the wealth of performance means and interpretation nuances. As proposed in our first hypothesis, the vast options exhibited with regards to tempo, rhythm, intonation, timbre, vibrato, bowings, fingerings and articulation indicate a most extensive diversity and are related to individual idiosyncrasies and personal imprints.

Previous eras have certainly witnessed heterogeneity and diversity of exegesis. Infinite ways of executing the tiniest of notes was reflected in each and every player’s individual intonation tendencies, timbre effects, vibrato stamping, dynamic shadings, tempo modifications, rhythmic alterations, and so on. However, being able to select from innumerable performance choices is what seems nowadays to be the current vogue, encouraging distinction and differentiation of the innermost performance parameters. No idiomatic effect, technical spectacle or musical novelty have remained pristine: a vast range of bowing effects, such as sul tasto, ricochet or spiccato, subtle variations of bow speed, pressure, and point of contact – all promote variegated dynamic and timbre shadings to the different textures; occasional use of the “swell” effect (messa di voce) alongside fastidious tailoring of vibrato, lends ample ring and enrichment to the sound; resonating open gut strings, accompanied by the use of period bow and low pitch, are presented side by side with forceful execution of chords and powerful tenuto articulation. Careful fidelity to the notes is still regarded as crucial, just as it always has, preventing exorbitance and entropy. So is the need for superb technical command of the instrument and staunch intonation. However, it is within the somewhat illusive borders of written notation and aesthetic conventions that a whole disarray of renditions is introduced to the fore.

As to the second hypothesis, findings indeed imply that currently active violinists incorporate 19th century performance devices while playing 19th century music: tempo modifications and agogic accents were detected among all players, although to different degrees. Scant use of vibrato was traced in the majority of recordings, used as an aid for phrase characterization and ambiance. Portamento was additionally found among various players, used as both an expressive and technical device (such as when it occurred during audible position shifts). Harmonics were used, although quite sparingly, and so was the manifestation of a wide palette of articulation, including the use of springy, flexible strokes, and (to a lesser degree) broad tenuto bowings.

As to the third of our hypothesis, deviation was indeed detected between the interpretations made to early (i.e., Beethoven sonata) and late (i.e., Brahms sonata) repertoire with regards to bowings, fingerings and articulation: wider usage of first position, open strings, covert position shifts and a relatively small-scale usage of varied articulation was found among the Beethoven players, whereas wider exhibition of portamento and more ample manifestation of articulation features was traced among their Brahms performance peers. Such divergence could be pertained to the influence of pre-1800 performance attributes on early 19th century repertoire players, brought about due to their adjoining historical periods. The somewhat cleaner and unsullied readings of the Beethoven sonata could also betoken Classical style characteristics, typified by structure formalism and clear texture, whereas the wide variance traced in the Brahms renderings denote Romantic expressivity and emotional exuberance.

Notwithstanding, attempting to label performers according to their degree of adhering to any one style or tradition seems quite elusive: performers might bring about assumed period features as portamento or harmonics on the one hand, yet display “irrelevant” parameters as strict rhythmic readings or extensive bowing changes on the other. While performance parameters, such as tempo and rhythmic alterations, vibrato manifestation or fingerings, seem significantly related to willful style objectives, individual signature and idiosyncrasies easily blur pre-designed conceptions.

Examining our fourth hypothesis, just a few pre-1800 performance features were observed as predominating HIP violinists’ renderings of the Beethoven more so than their MS peers. These include the more extensive implementation of harmonics, the wider preserve of originally notated bowings and, to a certain extent, the use of narrow vibrato. No statistical significance has been traced between both groups in the manner of execution of dynamics, overall tempo, rhythmic and tempo alterations, multiple-stops progression or intonation, whereas the higher degree of narrow vibrato utilization traced among the HIP players approached near significance, hence could not be unequivocally verified. As mentioned, the limited sample size requires that comprehensive conclusions be taken with care. Yet in general, a clear picture arises, suggesting an overall similarity in much of the parameters inspected as well as in the diversity of performance features rendered optional.

Faced with such diverse interpretations, and acknowledging the extensive mixture of HIP and MS stylistic approaches, it seems that with respect to C19th music the very distinction made between the two schools could be regarded as outdated when it comes to the current state of affairs. As is evident, manifestation of Messa di Voce, utilization of sparse vibrato, the penchant for first position, open strings, unnoticeable position shifts, habitual execution of multiple stops in a soft, light, and ‘airy’ manner or the compliance to the notated bowings and articulation were found amongst most performers of the Beethoven sonata. Moreover, different choices of dynamics and tempo, dissimilar pronunciations of inner tempo modifications and agogic accents, and divergence with regards to intonation tendencies or timbre shadings were traced regardless of a violinist’s presumed association to the HIP style.

The overwhelming impact of HIP ideals on the new generation of players could be well observed by studying the various renditions made to the Brahms sonata. On the whole, one could perhaps consider some players (e.g., Kaler, Goko, Yano) as presenting a somewhat ‘mainstream’ stylistic approach due to their general observance of rhythmic values, steadfast intonation, and an overall feeling of long-range ordination. However, such edict seems somewhat dogmatic in light of the varying extent of congruence to late 19th century performance featured by all, and especially considering the wide palette of interpretations featured by each and every one of the violinists examined.

Overall listening to the recordings at hand reveals that no one performance seems to exhibit the overwhelming impression of tempo and rhythmic instability, the seeming evenness of the dynamics spectrum or the rather ‘dry’ and ‘dim’ sound manifested in early recordings made at the beginning of the last century. However, several period features, such as Joachim’s use of finger vibrato in his playing of Brahms, his extensive use of portamento and audible shifts, or the utilization of a somewhat ‘tight,’ narrow ranged vibrato by his pupils, could certainly be observed. Even if no deliberate effort was made to adhere to the precise features found with regards to late 19th century Berlin violin school aesthetics, the variegation of performance practices engendered by the impact of HIP has certainly left its mark on most, if not all, violinists analyzed.

Such conclusions should not be surprising: engaging with HIP specialists throughout a performer’s numerous years of instrumental training has long been considered a standard route before launching a career. This, coupled with the almost exclusive supremacy of HIP recordings in the early music arena, makes it impossible these days to be unaware of HIP performance agenda. Unlike previous decades, we may have now come to the point where divaricating between ‘historically informed’ and ‘modern’ performances seems irrelevant: when it comes to current performance trends, complying with HIP principles in regards the quest for reconstruction of historical performance aesthetics has no pertinence to school affiliation, and has long ceased to represent affinity to any one specific circle or compositional period.

Observed from a broad perspective, it seems that the recording period under investigation could best be typified as an era of ‘over-choice’ environment: a new, broad range of performance platforms and an over-inflated music industry – among which social media, CDs, DVDs, MP3s or live concerts are but a few representatives – forces musicians to find new strategies for yielding top interpretations that surpass convention and strike an impact. Cooperative digital technologies, where home recording studios or even a simple cellular phone makes it possible for just about anyone to state his craft, enlarge the marketing framework and increase competition.

Seen in this context, the diversity in style, coupled with a vigorous thrust for innovation, discloses a more flexible approach to early music performance principles against the somewhat dogmatic adherence to ‘historical informed’ practices discerned in the past. Both MS and HIP aesthetics could now serve as an available assortment of interpretive options used to accomplish a peculiar, original rendering. What we face here, and rather unsurprisingly so, is a somewhat postmodern conception that puts pluralism, experimentalism, eclecticism, and elimination of hierarchical classifications into the forefront. In a world of fierce competition, challenging traditional boundaries and redefining established contexts for the sake of eminence seems most fitting.

### Implications and Future Direction

The first major contribution of the present research is that it provides empirical data on present-day violin recordings of 19th century repertoire, and on the impact of HIP ideals on current violinists performing 19th century music. This information is important given that, to the best of our knowledge, no analysis has been made of such repertoire with such a subject group: the relatively few studies focused on present-day violinists were mainly directed at the analysis of recordings made of J. S. Bach’s solo violin set. Recounting the current performance vogue will allow musicologists and performers to examine the influence of historical performance scholarship on professional performance and shed new light on the complex nature of style and period trends.

A second important implication of our study derives from the novel method of investigation involving computer analysis tools that have rarely been used in previous literature: selecting rich polyphonic music for investigation demanded a series of post-processing steps targeted at the extraction of the violin part from its piano accompaniment. Such procedure, enabling meticulous scrutiny made to features quite seldom dissected in previous analysis of violin concerted music (e.g., timbre spectrum, intonation profile) might assist researchers interested in recording analysis of repertoire of varied texture and orchestration.

Future research should be directed at expanding the scope of data to include recordings made of various other 19th century pieces. Investigation could deepen our knowledge of varied aspects related to performance, such as the relation between idiosyncratic trends and period style, interaction of performers and scholars, or the music industry’s impact on period performance conventions. Possible inquiries might well embark upon the confinements latent in current violin performance albeit the seeming illimitable possibilities of interpretation, on the probability of style-delineation within a context of ‘over-choice’ and peculiarity, on the influence of performance aesthetics of ‘remote’ musical styles (such as Jazz, Rock or non-western genres) on classical violinists’ vernacular, or on the course of studies taken by present-day violinists that could enable such an ample vocabulary.

## Author Contributions

EO serves as first and main contributing author. SC was in charge of the computerized analysis.

## Conflict of Interest Statement

The authors declare that the research was conducted in the absence of any commercial or financial relationships that could be construed as a potential conflict of interest.

## Footnotes

1 The year 1898 marks one of the very first commercial violin recordings, initiated by Dora Valesca – Becker and Henri Marteau under Bettini Cylinder. See Creighton (1975).

2 http://c19hip.web.ox.ac.uk/home

3 Measurements were made in linearly spaced time with values in Decibel sound pressure level (dB SPL). Values were related to 210-5 Pascal, which is the normative auditory threshold for a 1000-Hz sine wave.

4 Analysis was made for four notes of the segment (i.e., A5, F#5, G5, B5). Intensity measurements were taken at different points and included the note’s total duration, its deviation value, and points of departure and arrival (made in order to establish whether an increase or decrease in intensity was displayed). Violinists exhibiting increased intensity within three notes and above were regarded as notably implementing Messa di Voce.

5 Examination was based on comparisons of each player’s average BPM value to the overall group’s mean score.

6 Analysis procedure included the calculation of both individual and mean bar duration within the excerpt, followed by a subtraction of the maximum and minimum duration score from the mean score. This was followed by a deduction of the maximum – mean score from the minimum – mean score, resulting in each player’s discrepancy value’s point of reference (DV), followed by a calculation of the mean DV of the entire group of performers. Violinists exhibiting a higher than average DV score (i.e., found shortening or lengthening the bars more than the group mean discrepancy value) were regarded as exhibiting notable use of tempo modifications.

7 Analysis procedure included the calculation of both individual and mean eight-note duration within the excerpt, followed by a subtraction of the maximum and minimum duration score from the mean score. This was followed by a deduction of the maximum – mean score from the minimum – mean score, resulting in each player’s discrepancy value’s point of reference (DV), followed by a calculation of the mean DV of the entire group of performers. Violinists exhibiting a higher than average DV score (i.e., found shortening or lengthening the eight-notes more than the group mean discrepancy value) were regarded as exhibiting notable use of agogic accents.

8 The term ‘arpeggio’ refers to a swift bow stroke ‘spreading’ each note of the broken chord. The term ‘ricochet’ refers to a bouncing bow stroke in which the bow is dropped or thrown on the string and allowed to rebound and bounce upon playing each note of the broken chord.

9 Violinists exhibiting either arpeggio or ricochet were regarded as aligned with HIP style.

10 Analysis was conducted for four notes of the segment (i.e., A5, F#5, G5, B5). Pitch discrimination level was pre-determined as 10 cents and above. Violinists exhibiting deviation lower than absolute frequency value within three notes or above were regarded as exhibiting notable use of lower deviation from the absolute frequency value.

11 Vibrato extent is expressed in unit cent: 1200 log_2_ (peak/valley).

12 Analysis was conducted for four notes of the segment (i.e., A5, F#5, G5, B5). Procedure included vibrato-depth (i.e., extent) calculation of both individual notes and mean within the excerpt, followed by a subtraction of the maximum and minimum depth score from the mean score. This was followed by a deduction of the maximum – mean score from the minimum – mean score, resulting in each player’s discrepancy value’s point of reference (DV), followed by a calculation of the mean DV of the entire group of performers. Violinists exhibiting a smaller depth value than the average DV score (i.e., found narrowing their vibrato more than the group’s mean discrepancy value) were regarded as exhibiting notable use of narrow vibrato.
